# Interaction of *Ziziphus mucronata subsp. mucronata* Methanol Extract and First-Line Antibiotics is Synergistic *In Vitro* through Production of Reactive Oxygen Species

**DOI:** 10.1155/2020/4087394

**Published:** 2020-04-08

**Authors:** Aderonke Ariyike Olajuyigbe, Olufunmiso Olusola Olajuyigbe, Roger Murugas Coopoosamy

**Affiliations:** ^1^Department of Biochemistry, Olabisi Onabanjo University, Ago-Iwoye, Ogun State, Nigeria; ^2^Department of Microbiology, School of Science and Technology, Babcock University, PMB 4005, Ilisan-Remo, Ogun State, Nigeria; ^3^Department of Nature Conservation, Faculty of Natural Science, Mangosuthu University of Technology, Durban 4026, South Africa

## Abstract

With the increased incidence of antibacterial resistance in microorganisms, combining natural products from plants with antibiotics may be considered interesting alternatives for synergy to attain multitarget effects. In this study, the antioxidant activity of the methanol extract of *Ziziphus mucronata* and its interactions with antibiotics against bacteria of clinical importance were investigated. While its phytochemicals and antioxidant activities were determined by free radical scavenging assays, the antibacterial activities of the extract and its interactions with the antibiotics were determined by macrobroth dilution and the checkerboard methods. From the results, total phenolic content was 29.67 ± 1.90 mg GAE/100 g, total flavonoid content was 8.72 ± 0.08 mg QE/100 g, and total proanthocyanidin content was 1.94 ± 0.00 mg CE/100 g of dry plant material. The inhibition concentration 50% (IC_50_) of DPPH, BHT, and ascorbic acid was equal to 0.04 ± 0.02 mg/ml, respectively. Those of the ABTS, BHT, and ascorbic acid were equal to 0.02 ± 0.02, 0.04 ± 0.03, and 0.04 ± 0.02 mg/ml, respectively. The checkerboard assay showed that combining the extract with different antibiotics resulted in synergistic (38.75%), indifferent (30%), additive (28.75%), and antagonistic (2.5%) interactions. The interactions between the extract and antibiotics resulting in enhanced antibacterial activities could have resulted from the antioxidant activities of the extract mopping up the ROS generated by the antibiotics or the ability of both extract and antibiotics simultaneously producing reactive oxygen species with deleterious effects resulting in synergistic antibacterial effects.

## 1. Introduction

Forming the basis for practicing sophisticated ethnomedicine and providing excellent leads for new drug developments [1], the therapeutic significance of medicinal plants has become a popularized knowledge well disseminated by virtue of their use in the treatment of microbial infections [2]. While the medicinal properties of many plants have been reported [3] and pharmacological activities are due to the bioactive compounds present in them [4], the therapeutic failures of the drugs available today, the scarcity of novel antibiotics [5], emergence of resistant pathogens, adverse effects and limited spectrum of action of the currently available drugs [6], and high level of toxicity and carcinogenicity associated with synthetic antioxidants such as butylated hydroxytoluene (BHT) and *tert*-butyl hydroquinone (TBHQ) [7] have propelled the need to focus attention on discovering new and better antimicrobial and antioxidant agents of plant origin.

Furthermore, natural products from the plant are considered interesting alternatives as a result of increased incidence of antibiotic resistance [8]. Many plants have been evaluated for antimicrobial and their resistance-modifying activities [9]. Multitarget effects have been achieved by synergistic effects of combining extracts with antibiotics [10]. These drug-herbal combinations have improved the effectiveness of chemotherapeutic drugs with minimal toxicity to normal cells [11] and antibiotics having no intrinsic antibacterial activity as well as susceptibility of bacteria to previously ineffective antibiotics [12]. Although medicinal plants possess phytoconstituents effective against infections currently difficult to treat, their roles in disease treatments have been attributed to the antioxidant properties of these bioactive compounds in the plant [13]. Many plants containing free radical scavenging phenolic compounds react with catalytic metals and free radicals and scavenge oxygen to protect the biological system against deleterious effects of oxidative reactions produced by reactive oxygen species (ROS). However, while many phenolics have been known for their antioxidant and antimicrobial activities, many plants have remarkably combined with antibiotics to show varied degrees of interactions [14, 15] for which mechanisms of action are yet to be determined.

*Ziziphus mucronata subsp. mucronata*, commonly known as buffalo thorn, is a small to medium-sized tree. The plant can be identified during the winter months by the presence of its berry-like fruit ripening from March to September. During the flowering months, nectar-loving creatures visit these flowers in search of nectar. While its stem bark and roots are used for the treatment of rheumatism, syphilis, gonorrhea, gastrointestinal disorders such as dysentery and diarrhoea, and snake bites [16, 17], various parts of the plant are used for medicinal purposes [18]. To further establish the therapeutic potential of this plant species, this study investigated the phytochemicals, antioxidant potentials, and the influence of the methanol extract of *Ziziphus mucronata subsp. mucronata* on the antibacterial activities of some antibiotics against different bacterial species *in vitro* to indicate the possible effects of ROS produced as a result of combining the extract with the antibiotics.

## 2. Materials and Methods

### 2.1. Collection and Treatment of Plant Material

The stem bark of *Z*. *mucronata subsp. mucronata* were collected from the University of Fort Hare campus in Alice, air-dried at room temperature, authenticated by Prof. D.S. Grierson, and pulverized with a milling machine. One hundred grams of the pulverized sample was extracted with 500 ml of methanol for 72 h with shaking. The extract was filtered with Whatman No. 1 filter paper and concentrated under reduced pressure at 40°C using a rotary evaporator. After the extraction, the crude extract was redissolved in the extracting solvent to the required concentration for bioassay analysis. A voucher specimen (OLAJ/2010/ZM/01) was prepared and deposited in the Griffin's Herbarium of the University.

### 2.2. Chemicals and Reagents Used

All chemicals used—2,2′-azinobis-3-ethylbenzothiazoline-6-sulfonic acid (ABTS) diammonium salt, 1,1-diphenyl-2-picrylhydrazyl (DPPH), butylated hydroxytoluene (BHT), gallic acid, rutin, ascorbic acid (VC), quercetin and FeCl_3_, vanillin, Folin–Ciocalteu phenol reagent, and sodium carbonate—and the solvents were of analytical grade. Antibiotic powders of amoxicillin (AMX), chloramphenicol (CHL), ciprofloxacin (CIP), erythromycin (ERY), tetracycline hydrochloride (TET), metronidazole (MET), kanamycin (KAN), and nalidixic acid (NAL) were prepared and used according to the manufacturers' instructions.

### 2.3. Bacterial Strain

*Staphylococcus aureus* ATCC 6538, *Enterococcus faecalis* ATCC 29212, *Escherichia coli* ATCC 25922, *Enterobacter cloacae* ATCC 13047, *Klebsiella pneumoniae* ATCC 10031, *Proteus vulgaris* ATCC 6830, *Shigella sonnei* ATCC 29930, *Bacillus subtilis* KZN, *Proteus vulgaris* KZN, and *Enterococcus faecalis* KZN were used in this study. They were obtained from the Department of Biochemistry and Microbiology, University of Fort Hare, Alice, South Africa. The antibacterial assays were carried out using Mueller-Hinton II Agar (MHA) (Biolab) and broth. The inocula of the test bacteria were prepared using the colony suspension method [19]. Colonies picked from overnight cultures grown on nutrient agar were used to make suspensions of the test organisms in saline solution to give an optical density of approximately 0.1 at 600 nm. The suspension was then diluted 1 : 100 by transferring 0.1 mL of the bacterial suspension to 9.9 ml of sterile nutrient broth before being used.

#### 2.3.1. Determination of Total Flavonoids

Total flavonoids were estimated using the method of Marinova et al. [20]. Here, 0.5 ml of 2% AlCl_3_ ethanol solution was added to 0.5 mL of extract and allowed to stand for 60 min at room temperature before the absorbance was measured at 420 nm using an AJI-C03 UV-VIS spectrophotometer.

#### 2.3.2. Determination of Total Phenols

The total phenolic content of ZMM was determined by the modified Folin–Ciocalteu method [21]. Here, the extract (1 mg/mL) was mixed with 5 mL of Folin–Ciocalteu reagent (previously diluted with distilled water 1 : 10 v/v) and 4 mL (75 g/L) of sodium carbonate. The mixture was vortexed for 15 s and allowed to stand for 30 min at 40^o^C for colour to develop. The absorbance was measured in triplicate at 765 nm using an AJI-C03 UV-VIS spectrophotometer.

#### 2.3.3. Determination of Total Proanthocyanidins

The proanthocyanidin content of ZMM was determined by the modified method of Sun et al. [22]. A volume of 0.5 mL of 0.1 mg/mL of the extract solution was mixed with 3 ml of 4% vanillin-methanol solution and 1.5 ml hydrochloric acid. The mixture was allowed to stand for 15 min while the absorbance was measured at 500 nm using AJI-C03 UV-VIS spectrophotometer.

#### 2.3.4. Determination of Ferric Reducing Power

A modified spectrophotometric method of Ferreira et al. [23] was used for the measurement of reducing power of ZMM. The different concentrations of the extracts and the standards, rutin, and BHT (0.02–0.10 mg/mL; 1 mL) were mixed with 2.5 mL of 0.2 M phosphate buffer (pH 6.6) and 2.5 mL of potassium ferricyanide (K_3_Fe(CN)_6_) (1% w/v). The mixture was incubated at 50^o^C for 20 min after which 2.5 mL of trichloroacetic acid (TCA) (10% w/v) was added to the mixture before being centrifuged at 1000 rpm for 10 min. The supernatant of the mixture (2.5 mL) was then mixed with 2.5 mL of distilled water and 0.5 mL of 0.1% w/v FeCl_3_. The absorbance was measured at 700 nm in a AJI-C03 UV-VIS spectrophotometer.

#### 2.3.5. DPPH Radical Scavenging Assay

To determine the free radical scavenging activity of ZMM against DPPH, the method of Liyana-Pathirana and Shahidi [24] was adopted. One milliliter of 0.135 mM DPPH in methanol was mixed with 1 mL of different concentrations (0.02–0.1 mg/mL) of ZMM. The reaction mixture was vortexed thoroughly and left in the dark at room temperature for 30 min. Ascorbic acid and butylated hydroxytoluene (BHT) were used as reference standards while methanol was used as control. Reduction of the stable DPPH radical was used as a marker of antioxidant capacity of ZMM. The free radical scavenging activity of the extract indicated by changes in colour from deep-violet to light-yellow was measured using AJI-C03 UV-VIS spectrophotometer at 517 nm.

#### 2.3.6. ABTS Radical Scavenging Assay

For ABTS radical scavenging activity of the methanol extract, the modified method of Johnstone et al. [25] was adopted. The working concentration containing equal volumes of 7 mM ABTS solution and 2.4 mM potassium persulfate solution was prepared and allowed to react for 12 h at room temperature in a dark cabinet. The resulting solution was further diluted by mixing 1 mL ABTS^+^ solution with 60 ml of methanol to obtain an absorbance of 0.708 ± 0.001 units at 734 nm using AJI-C03 UV-VIS spectrophotometer.

#### 2.3.7. Determination of Minimal Inhibitory Concentration (MIC)

The minimum inhibitory concentrations (MICs) for ZMM and the antibiotics were determined in duplicate by the macrobroth dilution method in Mueller-Hinton broth (MHB) according to CLSI (Clinical Laboratory Standardization Institute) [26]. To determine the MICs of each antibiotic, the concentrations prepared for each of AMX, TET, MET, NAL, KAN, and CHL ranged between 0.224 and 500 *μ*g/mL. While CIP concentration ranged between 0.005 and 5 *μ*g/mL, the concentration of ERY was between 0.049 and 50 *μ*g/mL and that of ZMM was between 4.88 and 5000 *μ*g/mL. The antibiotic concentrations were prepared by serial dilution in double-strength MHB. To determine their combinatorial effects, combinations of different concentrations ranging from 1/2 X MIC to 8 X MIC of each antibiotic and those of the extract were prepared in double-strength MHB. Each tube was inoculated with 100 *µ*L of each of the bacterial strains. Blank Mueller-Hinton broth was used as a negative control. The bacterial containing tubes were incubated at 37°C for 24 h. The MIC was defined as the lowest concentration that showed no growth in the Mueller-Hinton broth. Each combination assay was performed in duplicate.

### 2.4. Checkerboard Assay

The interactions between the extract and the antibiotics were determined using the checkerboard assay as previously described [27]. The fractional inhibitory concentration (FIC) indices were calculated using the formula: FIC index = (MIC of extract in combination/MIC of extract alone) + (MIC of antibiotics in combination/MIC of antibiotics alone). In antimicrobial combination, Petersen et al. [27] defined synergy as ∑FIC ≤ 0.5, additivity as 5 < ∑FIC ≤ 1, indifference as 1 < ∑FIC ≤ 4, and antagonism as ∑FIC > 4.

### 2.5. Statistical Analysis

Data were expressed as means ± standard deviations (SDs) of three replicate determinations and then analyzed by SPSS V.16 (Statistical Program for Social Sciences, SPSS Corporation, Chicago, IL). One-way analysis of variance (ANOVA) and Duncan's new multiple-range test were used to determine the differences among the means. *p* values < 0.05 were regarded to be significant. The Pearson correlation analysis was performed between antioxidant activity and total phenolic content.

## 3. Results

**Figure 1:**
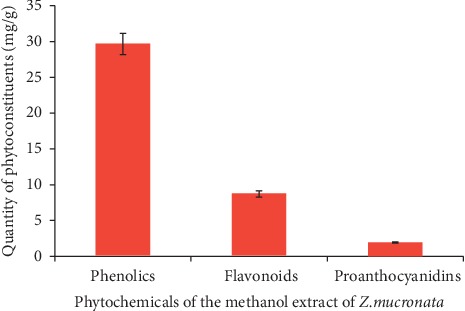
Quantity (mean ± SD) of phytochemicals in the methanol extract of *Z. mucronata*.

In this study, the quantity of total phenolic content, flavonoids, and proanthocyanidins in the methanol extract of *Z. mucronata* (ZMM) is presented in Figure 1. The total phenolic content of ZMM was 29.67 ± 1.901 mg GAE/100 g of the dry weight of plant material. The total flavonoid content was 8.72 ± 0.076 mg QE/100 g of dry plant material. The total proanthocyanidin content was 1.94 ± 0.004 mg CE/100 g of dry plant material.

The absorbance of the reducing ability of ZMM determined by FRAP method was measured with spectrophotometer at 700 nm as shown in Table 1. The ferric reducing activity of ZMM was significantly lower than those of the standard drugs, but a gradual increase in concentrations of this extract increased its reducing power capability significantly. The dose-dependent reducing potentials of this extract indicated that there are antioxidant compounds with electron donating ability in *Z. mucronata*.

The free radical scavenging or hydrogen donor potentials and evaluation of the antioxidative activity of medicinal plant extracts have been widely tested with DPPH radical [28] and are often determined using the percentage inhibition of DPPH and IC_50_ of the extract [29]. The higher the percent inhibition of DPPH and the lower the IC_50_ value, the higher the free radical scavenging ability/antioxidant power of the medicinal plant. In this study, the concentration-dependent percentage inhibition of DPPH free radicals by the extract and BHT and ascorbic acid are as shown in Table 2. The *in vitro* 50% inhibition concentration (IC_50_) values obtained for the DPPH inhibition of ZMM, BHT, and ascorbic acid were found to be 0.043 ± 0.02, 0.042 ± 0.03, and 0.040 ± 0.02 mg/mL, respectively.

The effect of ZMM, BHT, and ascorbic acid on ABTS radical cation scavenging activity is presented in Table 3. While the IC_50_ of ZMM was 0.023 ± 0.02 mg/ml and was significantly different from those of BHT (0.041 ± 0.03 mg/mL) and ascorbic acid (0.042 ± 0.02 mg/mL), those of BHT and ascorbic acid were not significantly different from each other. The lower IC_50_ of the extract showed that it possesses stronger radical scavenging activity than BHT and ascorbic acid used as controls.

From the macrobroth dilution, ZMM and the antibiotics exerted a varied degree of inhibitory and interactions against the test organisms. While the extract had minimum inhibitory concentrations (MICs) ranged between 141.82 and 568.18 *µ*g/mL, those of CIP (0.018 and 0.284 *µ*g/mL), ERY (0.089 and 22.73 *µ*g/mL), TET (0.44 and 14.20 *µ*g/mL), CHL (0.89 and 28.41 *µ*g/mL), AMX (0.89 and 454.55 *µ*g/mL), NAL (1.78 and 56.82 *µ*g/mL), KAN (1.78 and 454.55 *µ*g/mL), and MET (14.20 and 113.64 *µ*g/mL) varied. The MICs of ZMM are higher than those of the different antibiotics. According to the MIC breakpoints recommended by EUCAST [19] and BSAC [30], strains of *Enterococcus* species, *Enterobacter* species, *Staphylococcus* species, and Gram-positive aerobes having MIC values of ≤0.25 *µ*g/mL for ERY, ≤0.5 *µ*g/mL for CIP, ≤1 *µ*g/mL for TET, ≤4 *µ*g/mL for MET, ≤4 *µ*g/mL for AMX, ≤8 *µ*g/mL for KAN, ≤ 8 *µ*g/mL for CHL, and ≤16 *µ*g/mL for NAL are classified as being susceptible. Based on MIC breakpoints for the antibiotics as indicated by EUCAST [19] and BSAC [30], the MIC breakpoint showed that all isolates were susceptible to CIP, all were resistant to MET, all isolates were susceptible to CHL with the exception of *E. faecalis* KZN, *S. sonnei* ATCC 29930, *S. aureus* OK_2a_, and *K. pneumoniae* ATCC 10031 which were susceptible to ERY, *K. pneumoniae* ATCC 10031, *B. subtilis* KZN, and *S. aureus* OK_2a_ which were susceptible to TET, *K. pneumoniae* ATCC 10031, *P. vulgaris* KZN, and *E. faecalis* KZN which were susceptible to AMX, *S. aureus* ATCC 6538, *B. subtilis* KZN, and *P. vulgaris* KZN which were susceptible to KAN while *K. pneumoniae* ATCC 10031, *P. vulgaris* ATCC 6830, *B. subtilis* KZN, and *S. sonnei* ATCC 29930 were susceptible to NAL. Combining TET with ZMM resulted in the reduction of MICs of the ZMM to concentrations ranging between 0.0.0005 and 284.10 *µ*g/mL while that of the TET ranged between 0.028 and 14.20 *µ*g/mL. AMX combined with ZMM resulted in the MICs of the ZMM being reduced to concentrations ranging between 0.0005 and 568.18 *µ*g/mL while that of the AMX was reduced to concentrations ranging between 0.03 and 14.20 *µ*g/mL. *Bacillus subtilis* KZN was not affected by the herbal-drug combination. ERY combined with ZMM resulted in the reduction of the concentrations of the ZMM to concentrations between 0.028 and 568.18 *µ*g/mL and those of ERY to concentrations ranging between 0.003 and 568.18 *µ*g/mL, with the exception of *P. vulgaris* ATCC 6830 and *S. aureus* ATCC 6538 not affected by ERY combined with the ZMM. CIP combined with ZMM resulted in the reduction of the MICs of the ZMM to concentrations ranging between 8.88 and 284.1 *µ*g/mL while those of CIP were reduced to concentrations ranging between 0.018 and 0.0.284 *µ*g/mL. While the combination of NAL with ZMM did not result in a significant reduction in the MICs of the ZMM against most of the isolates, CHL reduced the MICs of ZMM significantly. Combining CHL with ZMM resulted in MICs of the ZMM ranging between 7.10 and 284.10 *µ*g/mL and those of CHL ranging 0.44 and 14.20 *µ*g/mL. With the exception of *P. vulgaris* KZN and *E. faecalis* KZN to which the combination of KAN and ZMM had no effect, KAN reduced the MICs of the ZMM to concentrations ranging between 7.10 and 568.18 *µ*g/mL and that of this antibiotic was reduced to 0.444 and 28.41 *µ*g/mL. Generally, the combination of the antibiotics with the extract resulted in reduction in the MICs of antibiotics and the extract significantly as shown in Table 4.

The fractional inhibitory concentration (FIC) index of the ZMM combined with each of the antibiotics resulted in synergistic (38.75%), indifferent (30%), additive (28.75%), and antagonistic interactions (2.5%). While the fractional inhibitory concentration index (FICI) for the synergistic interaction was between 0.0004 and 0.50, the FICI for the additive interaction was between 0.531 and 1.0, that of indifference was between 1.063 and 2.5, and that of antagonistic interaction was between 3.0 and 18 (Table 5).

## 4. Discussion

In medicinal plants, pharmacological activities of extracts are due to polyphenolic compounds such as alkaloids, flavonoids, and phenolic compounds. The biological and pharmacological importance of these substances has been reported. Tannins possess antimicrobial activities able to damage bacterial membranes or delay bacterial growth for sufficient time for bacterial elimination and for the host to develop its immune system [31]. Flavonoids inhibited cytoplasmic membrane function, DNA gyrase, and *β*-hydroxyacyl-acyl carrier protein dehydratase activities [32] to inhibit microbial growth. Phenols have antioxidants, antibacterial, antiviral, anticancer, and anti-inflammatory properties [33]. The pharmacological activity of the polyphenols is mainly due to their redox properties allowing them to act as reducing agents, hydrogen donors, singlet oxygen quenchers, metal chelators, and reductants of ferryl haemoglobin [34]. In this study, the amount of DPPH scavenging activity of the methanol extract is dependent on the concentration of the phenolic content of the extract. The strong DPPH scavenging activity of the extracts could be attributed to their catechins and some low molecular polyphenols [35] and the number of aromatic rings and nature of hydroxyl group's substitution [36].

In treating microbial infections, while oxidative stress in bacteria caused by xenobiotics [37–39] produced toxic effects, a number of antibiotics including quinolones [40, 41], aminoglycosides [42], rifampicin [43], and chloramphenicol [44] were known to induce production of reactive oxygen species (ROS) in different bacterial cells regardless of their specific targets [45]. During the oxidation process, active oxygen species produced by cells are affected by different chemicals during redox cycling [46]. Gyulkhandanyan et al. [47] reported that this catalytically produced oxidative stress from the redox cycle is a possible mode of action of antibiotics. Though the antibacterial activities of the antibiotics depend on ROS produced and medicinal plants, though having antioxidant and antibacterial activities, the synergy resulting from combining the antibiotics and the extract could have resulted from the antibacterial effects of ROS produced by them in the bacterial species. The ROS from the antibiotics and, possibly, those from the extract could have affected different target sites in the bacteria to exert their antibacterial activity. If the damaging effects of the ROS produced by the antibiotics were more than the antioxidant defense activity of the extract, the bacterial defense mechanism could have been reduced to allow the antibiotics to effectively reach its target sites. The synergy could, therefore, have been in response to less reactive but longer-lived and more stable free radicals produced from the reaction between antioxidants in the extract and the free radicals generated by the antibiotics against the bacterial isolates.

On the other hand, since antioxidants defend physiologically active system against ROS and polyphenols could take part in the generation of ROS and act as prooxidants [48], the extract with high antioxidative effects could have prevented the ROS generated by antibiotics from exerting antibacterial effects on the bacteria. In previous studies, Desesso et al. [49] reported that only nonspecific scavengers having low redox potential could protect against bacterial susceptibility to antibiotics. ROS scavengers, such as glutathione and ascorbic acid, prevented the susceptibility of ciprofloxacin-sensitive *Escherichia coli* MG1655*, Enterococcus faecalis*, and *Staphylococcus aureus* [50]. They confer protection against fluoroquinolones and aminoglycosides [51] but augment the antibacterial activity of *β*-lactams against *E. coli* [52]. Although increase in ROS could result in the induction of mutagens and bacterial resistance [42] and polyphenols protect bacterial cells against ciprofloxacin toxicity [53], the ROS generated from the combined antibiotics and extract could have resulted in reduced bacterial susceptibility in some of the isolates as the phytochemicals could have protected the bacterial cells against the effectiveness of each antibiotic as indicated by the susceptibility resulting in the varied degree of additive, indifference, and antagonistic interactions from the various combinations.

## 5. Conclusion

There were varied degrees of interactions between ZMM and different antibiotics commonly used in the treatment of microbial infections. The antibacterial effects resulting from different synergistic interaction between ZMM and the antibiotics could be attributed to their ability to produce ROS simultaneously to effect bactericidal action while antibacterial effects resulting from other interactions between ZMM and the antibiotics could be attributed to the ability of the extract to act as antioxidant mopping up the ROS generated by the antibiotics and vice versa against the bacterial isolates. The modulating effect of each of the phytochemicals on the susceptibility of each bacterial species to each antibiotic requires further investigations that may be of significant application in the treatment of bacterial infections in disease situations.

## Figures and Tables

**Table 1:** Mean ± SD of ferric reducing power of methanol extract of *Z. mucronata*.

	Ferric reducing power of methanol extract of *Z. mucronata*				
	0.02 mg/mL	0.04 mg/mL	0.06 mg/mL	0.08 mg/mL	0.10 mg/mL
ZMM	0.021 ± 0.002^e^	0.113 ± 0.001^d^	0.237 ± 0.001^c^	0.335 ± 0.002^b^	0.342 ± 0.002^a^
BHT	0.182 ± 0.001^e^	0.347 ± 0.001^d^	0.445 ± 0.002^c^	0.564 ± 0.001^b^	0.633 ± 0.001^a^
Ascorbic acid	0.275 ± 0.00^e^	0.562 ± 0.001^d^	0.752 ± 0.001^c^	0.837 ± 0.001^b^	1.218 ± 0.002^a^

Average ferric reducing power scavenging activities with different superscripts along the same row are significantly different (*p* < 0.05).

**Table 2:** DPPH inhibition (%) by the methanol extract of *Z. mucronata*.

	% inhibitions of DPPH ± SD at different concentrations of the extracts					
Conc.	0.02 mg/mL	0.04 mg/mL	0.06 mg/mL	0.08 mg/mL	0.1 mg/mL	IC_50_
ZMM	33.9 ± 0.03^e^	45.99 ± 0.02^d^	73.01 ± 0.02^c^	86.07 ± 0.04^b^	94.86 ± 0.02^a^	0.043 ± 0.02
BHT	38.96 ± 0.02^e^	47.2 ± 0.03^d^	75.86 ± 0.03^c^	91.92 ± 0.05^b^	97.78 ± 0.03^a^	0.042 ± 0.03
Ascorbic acid	25.23 ± 0.04^e^	49.56 ± 0.03^d^	72.23 ± 0.02^c^	94.77 ± 0.03^b^	96.89 ± 0.02^a^	0.040 ± 0.02

Average DPPH scavenging activities with different superscripts along the same row are significantly different (*p* < 0.05).

**Table 3:** ABTS inhibition (%) by the methanol extract of *Z. mucronata*.

	% inhibitions of ABTS^+^ ± SD at different concentrations of the extracts					
Conc.	0.02 mg/mL	0.04 mg/mL	0.06 mg/mL	0.08 mg/mL	0.1 mg/mL	IC_50_
ZMM	43.67 ± 0.02^e^	58.6 ± 0.02^d^	81.67 ± 0.03^c^	92.33 ± 0.03^b^	97.00 ± 0.02^a^	0.023 ± 0.02
BHT	34.96 ± 0.02^e^	49.2 ± 0.03^d^	81.86 ± 0.02^c^	91.92 ± 0.02^b^	97.78 ± 0.03^a^	0.041 ± 0.03
Ascorbic acid	35.23 ± 0.02^e^	47.56 ± 0.02^d^	78.23 ± 0.02^c^	94.77 ± 0.02^b^	96.89 ± 0.03^a^	0.042 ± 0.02

Average ABTS scavenging activities with different superscripts along the same row are significantly different (*p* < 0.05).

**Table 4:** Antibacterial and effects of combining methanolic extract of *Z. mucronata* with different antibiotics *in vitro*.

Organisms used	ZMM MIC (*µ*g/mL)	TET MIC (*µ*g/mL)	ZMM/TET MIC (*µ*g/mL + *µ*g/mL)	AMX MIC (*µ*g/mL)	ZMM/AMX MIC (*µ*g/mL + *µ*g/mL)	MET MIC (*µ*g/mL)	ZMM/MET MIC (*µ*g/mL + *µ*g/mL)	ERY MIC (*µ*g/mL)	ZMM/ERY MIC (*µ*g/mL + *µ*g/mL)
*Staphylococcus aureus* ATCC 6538	568.18	7.10	284.1/14.20	28.41	284.1/14.20	14.20	284.1/14.20	5.68	568.1/5.68
*Enterococcus faecalis* ATCC 29212	568.18	3.55	141.82/7.10	7.10	71.02/1.78	28.41	568.18/28.41	2.84	17.73/0.18
*Klebsiella pneumoniae* ATCC 10031	568.18	0.44	0.05/0.03	1.78	35.51/0.89	28.41	141.82/7.10	0.089	0.28/0.003
*Proteus vulgaris* ATCC 6830	568.18	7.10	17.73/0.89	227.27	71.02/3.55	56.82	568.18/28.41	22.73	568.18/5.68
*Bacillus subtilis* KZN	568.18	1.78	35.51/0.89	56.82	568.18/28.41	28.41	284.1/14.20	5.68	284.1/2.84
*Proteus vulgaris* KZN	568.18	7.10	284.1/14.20	1.78	17.73/0.44	56.82	284.1/14.20	11.36	284.1/2.84
*Enterococcus faecalis* KZN	284.1	14.20	284.1/14.20	0.89	17.73/0.44	56.82	284.1/14.20	11.36	35.51/0.36
*Staphylococcus aureus* OK_2a_	284.1	0.89	0.0005/0.03	113.64	71.02/3.55	56.82	284.1/14.20	0.18	4.44/0.044
*Staphylococcus aureus* OK_2b_	568.18	1.78	17.73/0.89	14.20	71.02/3.55	113.64	284.1/14.20	11.36	284.1/2.84
*Shigella sonnei* ATCC 29930	141.82	1.78	0.05/0.03	454.55	0.05/0.03	56.82	71.02/3.55	0.36	0.28/0.003

Organisms used	ZMM MIC (*µ*g/mL)	CIP MIC (*µ*g/mL)	ZMM/CIP MIC (*µ*g/mL + *µ*g/mL)	NAL MIC (*µ*g/mL)	ZMM/NAL MIC (*µ*g/mL + *µ*g/mL)	KAN MIC (*µ*g/mL)	ZMM/KAN MIC (*µ*g/mL + *µ*g/mL)	CHL MIC (*µ*g/mL)	ZMM/CHL MIC (*µ*g/mL + *µ*g/mL)

*Staphylococcus aureus* ATCC 6538	568.18	0.018	71.02/0.002	28.41	284.1/14.20	1.78	35.51/0.44	1.78	7.10/3.55
*Enterococcus faecalis* ATCC 29212	568.18	0.284	141.82/0.142	28.41	568.18/28.41	113.63	284.1/14.20	1.78	35.51/1.78
*Klebsiella pneumoniae* ATCC 10031	568.18	0.071	35.51/0.018	3.55	71.02/3.55	14.20	17.73/3.55	1.78	8.88/0.44
*Proteus vulgaris* ATCC 6830	568.18	0.071	35.51/0.018	1.78	71.02/3.55	28.41	141.82/7.10	7.10	35.51/1.78
*Bacillus subtilis* KZN	568.18	0.018	8.88/0.005	7.10	71.02/3.55	3.55	7.10/2.84	3.55	35.51/1.78
*Proteus vulgaris* KZN	568.18	0.284	141.82/0.142	56.82	568.18/28.41	7.10	568.18/28.41	0.89	284.1/14.20
*Enterococcus faecalis* KZN	284.1	0.284	284.1/0.284	56.82	284.1/14.20	454.55	568.18/28.41	28.41	284.1/14.20
*Staphylococcus aureus* OK_2a_	284.1	0.071	71.02/0.002	28.41	284.1/14.20	14.20	17.73/3.55	7.10	17.73/0.89
*Staphylococcus aureus* OK_2b_	568.18	0.018	35.55/0.018	56.82	284.1/14.20	28.41	7.10/2.84	7.10	35.51/1.78
*Shigella sonnei* ATCC 29930	141.82	0.018	71.02/0.002	14.20	284.1/14.20	28.41	141.82/7.10	7.10	17.73/0.89

ADD = additive; ANT = antagonistic; IND = indifference; SYN = synergy; ZMM = methanolic extract of *Z. mucronata*; TET = tetracycline; AMX = amoxicillin; MET = metronidazole; ERY = erythromycin; KAN = kanamycin; NAL = nalidixic acid; CIP = ciprofloxacin; CHL = chloramphenicol; REM = remarks.

**Table 5:** Fractional inhibitory concentration indices of the antibacterial combinations.

	Fractional inhibitory concentration indices (FICI) of different antibiotics combined with methanolic extract of Z. mucronata															
Organisms used	ZMM/TET	REM	ZMM/AMX	REM	ZMM/MET	REM	ZMM/ERY	REM	ZMM/KAN	REM	ZMM/NAL	REM	ZMM/CIP	REM	ZMM/CHL	REM
*Staphylococcus aureus* ATCC 6538	2.50	IND	1.0	ADD	1.50	IND	2.00	IND	0.31	SYN	1.0	ADD	0.24	SYN	2.01	IND
*Enterococcus faecalis* ATCC 29212	2.25	IND	0.38	SYN	0.50	SYN	0.09	SYN	0.63	ADD	1.5	IND	0.75	ADD	1.06	IND
*Klebsiella pneumoniae* ATCC 10031	0.68	ADD	0.56	ADD	0.50	SYN	0.034	SYN	0.28	SYN	1.13	IND	0.32	SYN	0.26	SYN
*Proteus vulgaris* ATCC 6830	0.16	SYN	0.14	SYN	1.50	IND	1.25	IND	0.50	SYN	2.13	IND	0.32	SYN	0.31	SYN
*Bacillus subtilis* KZN	0.51	ADD	1.50	ADD	0.75	ADD	1.00	ADD	0.81	ADD	0.63	ADD	0.29	SYN	0.57	ADD
*Proteus vulgaris* KZN	2.50	IND	0.28	SYN	0.75	ADD	0.75	ADD	5.00	ANT	1.5	IND	1.00	ADD	16.46	ANT
*Enterococcus faecalis* KZN	2.00	IND	0.56	ADD	1.25	IND	0.16	SYN	2.06	IND	1.25	IND	2.00	IND	1.5	IND
*Staphylococcus aureus* OK_2a_	0.03	SYN	0.28	SYN	1.25	IND	0.26	SYN	0.31	SYN	1.50	IND	0.28	SYN	0.19	SYN
*Staphylococcus aureus* OK_2b_	0.53	ADD	0.37	SYN	0.62	ADD	0.75	ADD	0.11	SYN	0.75	ADD	1.06	IND	0.31	SYN
*Shigella sonnei* ATCC 29930	0.02	SYN	0.0004	SYN	0.56	ADD	0.01	SYN	1.25	IND	3.00	IND	0.61	ADD	0.25	SYN

ADD = additive; ANT = antagonistic; IND = indifference; SYN = synergy; ZMM = methanolic extract of *Z. mucronata*; TET = tetracycline; AMX = amoxicillin; MET = metronidazole; ERY = erythromycin; KAN = kanamycin; NAL = nalidixic acid; CIP = ciprofloxacin; CHL = chloramphenicol; REM = remarks.

## Data Availability

The data used to support the findings of this study are included within the article.
